# CCN2/CTGF promotor activity in the developing and adult mouse eye

**DOI:** 10.1007/s00441-020-03332-4

**Published:** 2021-01-29

**Authors:** Andrea E. Dillinger, Sabrina Kuespert, Franziska Froemel, Ernst R. Tamm, Rudolf Fuchshofer

**Affiliations:** Institute of Human Anatomy and Embryology, University of Regensburg, 93053 Regensburg, Germany

**Keywords:** Eye development, Trabecular meshwork, Glial lamina, Neural crest, Astrocyte

## Abstract

CCN2/CTGF is a matricellular protein that is known to enhance transforming growth factor-β signaling and to induce a myofibroblast-like phenotype in a variety of cell types. Here, we investigated *Ccn2/Ctgf* promotor activity during development and in the adult mouse eye, using *CTGF*^*LacZ/*+^ mice in which the β-galactosidase reporter gene *LacZ* had been inserted into the open reading frame of *Ccn2/Ctgf*. Promotor activity was assessed by staining for β-galactosidase activity and by immunolabeling using antibodies against β-galactosidase. Co-immunostaining using antibodies against glutamine synthetase, glial fibrillary acidic protein, choline acetyltransferase, and CD31 was applied to identify specific cell types. *Ccn2/Ctgf* promotor activity was intense in neural crest-derived cells differentiating to corneal stroma and endothelium, and to the stroma of choroid, iris, ciliary body, and the trabecular meshwork during development. In the adult eye, a persistent and very strong promotor activity was present in the trabecular meshwork outflow pathways. In addition, endothelial cells of Schlemm’s canal, and of retinal and choroidal vessels, retinal astrocytes, Müller glia, and starburst amacrine cells were stained. Very strong promoter activity was seen in the astrocytes of the glial lamina at the optic nerve head. We conclude that CCN2/CTGF signaling is involved in the processes that govern neural crest morphogenesis during ocular development. In the adult eye, CCN2/CTGF likely plays an important role for the trabecular meshwork outflow pathways and the glial lamina of the optic nerve head.

## Introduction

CCN2 is a member of the cellular communication network (CCN) family of extracellular matrix-associated and heparin-binding matricellular proteins (Brigstock 2003; Leask 2020; Perbal and Perbal 2016; Perbal 2013, 2018). It is more commonly known under its original name connective tissue growth factor (CTGF) that was recently discontinued by the HUGO Gene Nomenclature Committee which adopted the use of CCN2 instead (Perbal et al. 2018). Because of the fairly recent change and for the sake of clarity, we will continue to use the term CCN2/CTGF throughout this contribution. Like other members of the CCN family, CCN2/CTGF is structurally characterized by four conserved, cysteine-rich domains. The domains consist of the insulin-like growth factor binding protein (IGFBP) domain, the von Willebrand type C repeats (vWC) domain, the thrombospondin type 1 repeat (TSR) domain, and a C-terminal domain (CT) with a cysteine knot motif.

Studies in *Ccn2/Ctgf*-deficient mice show an important role of CCN2/CTGF for proper development of cartilage, vasculature, and pancreatic islets (Crawford et al. 2009; Hall-Glenn et al. 2012; Ivkovic et al. 2003). In the adult organism, a major function of CCN2/CTGF is the amplification of transforming growth factor-β (TGF-β) signaling by both activating latent TGF-β and enhancing its binding to the TGF-β receptor II (Abreu et al. 2002; Gressner et al. 2009; Khankan et al. 2011; Mori et al. 1999). TGF-β signaling is further augmented through the TGF-β-induced increase in *Ccn2/Ctgf* expression that has been consistently observed in many different cell types (Fuchshofer et al. 2009, 2007; Grotendorst et al. 1996; Holmes et al. 2001; Igarashi et al. 1993; Leask et al. 2001). CCN2/CTGF is highly overexpressed in conditions associated with increased extracellular matrix (ECM) synthesis and fibrosis such as scleroderma (Sato et al. 2000; Yamamoto et al. 2005), diabetic nephropathy and kidney fibrosis (Mason 2013; Yin and Liu 2019), fibrotic liver diseases (Gressner and Gressner 2008), idiopathic pulmonary fibrosis (Richeldi et al. 2020), or glaucoma (Browne et al. 2011; Ho et al. 2020; Wallace et al. 2013). In fibrotic conditions, CCN2/CTGF appears to be primarily involved in the differentiation of resident cells into myofibroblasts (Kapoor et al. 2008; Liu et al. 2010), a highly specialized cell type of variable origin that combines extracellular matrix synthesis with contractility (Bochaton-Piallat et al. 2016; Carthy 2018; Hinz et al. 2007, 2012).

The role of CCN2/CTGF in myofibroblast differentiation is also seen in normal cell types with myofibroblast-like characteristics such as the trabecular meshwork in the eye (Braunger et al. 2015a; Tamm et al. 2015). Depletion of CCN2/CTGF in trabecular meshwork cells causes a substantial loss of their actin cytoskeleton while its overexpression increases trabecular meshwork contractility and extracellular matrix production (Junglas et al. 2012). The increase in trabecular meshwork contractility causes dysfunction of the aqueous humor outflow pathways, high intraocular pressure and glaucoma (Junglas et al. 2012; Reinehr et al. 2019). In the present study, we wanted to learn more about the specific cell types that express CCN2/CTGF in the eye. Immunohistochemical studies are available that studied its localization in ocular tissues (Pi et al. 2011; van Setten et al. 2016). Still such studies offer only limited information about the cellular expression of *Ccn2/Ctgf* as it is a secreted molecule that binds with high affinity to heparan sulfate proteoglycans and other molecules on cellular surfaces. For this reason, cellular immunoreactivity for CCN2/CTGF might not necessarily indicate its cellular origin. We therefore studied *Ccn2/Ctgf* promotor activity in *CTGF*^*LacZ/*+^ mice in which the β-galactosidase marker gene *LacZ* has been inserted into the open reading frame of *Ccn2/Ctgf* (Crawford et al. 2009). Here, we report about distinct and hitherto not identified cellular sites of substantial *Ccn2/Ctgf* expression during ocular development and in the adult eye.

## Material and methods

### Animals

In *CTGF*^*LacZ/*+^ mice, the β-galactosidase marker gene *LacZ* was inserted into the open reading frame of *Ccn2/Ctgf* (Crawford et al. 2009). In this study, we used heterozygous *CTGF*^*LacZ/*+^ mice to investigate the distribution pattern of *Ccn2/Ctgf* promotor activity during eye development. *CTGF*^*LacZ/*+^ mice were found to be viable and fertile corroborating previous observations (Crawford et al. 2009). CTGF ^*LacZ/LacZ*^ animals were not included in this study as homozygous deficiency causes severe skeletal and vascular abnormalities leading to the death of the animals at birth (Ivkovic et al. 2003). We expected that *Ccn2*/*Ctgf* promoter activity under this condition would not necessarily reflect that of wild-type mice. Mice were housed under standardized conditions of 62% air humidity and 21 °C room temperature (RT). Feeding was ad libitum. Animals were kept at a 12 h light/dark cycle (6:00–18:00). All procedures conformed to the tenets of the National Institutes of Health Guidelines on the Care and Use of Animals in Research, the EU Directive 2010/63/E and the ARVO Statement for the Use of Animals in Ophthalmic and Vision Research, and were approved by the local authorities (54-2532.1-44/12; Regierung Oberpfalz, Bavaria, Germany). Before enucleation of the eyes, mice were anesthetized with CO_2_ and euthanized by atlanto-occipital dislocation.

### RNA analysis

Total RNA from sensory retinal tissue was extracted with TriFast™ (Peqlab, Erlangen, Germany) according to the manufacturer’s recommendations. cDNA was prepared from total RNA using the qScript™ cDNA Synthesis Kit (Quanta Biosciencies Gaithersburg, USA) according to the manufacturer’s instructions. Real-time RT-PCR was performed on a BioRad CFX Real-Time PCR Detection System (BioRad, Munich, Germany) with the temperature profile as follows: 50 cycles of 20 s melting at 94 °C, 10 s of annealing at 60 °C, and 20 s of extension at 60 °C. All primers were purchased from Invitrogen (Carlsbad, USA) and extended over exon–intron boundaries. CTGF forward 5′TGACCTGGAGGAAAACATTAAGA3′, CTGF reverse 5′AGCCCTGTATGTCTTCACACTG3′, RPL32 forward 5′GCTGCCATCTGTTTTACGG3′, RPL32 reverse 5′TGACTGGTGCCTGATGAACT3′. RNA that was not reverse transcribed served as negative control. For relative quantification of the experiments, RPL32 was used as a housekeeping gene. Quantification was performed using BioRad CFX Manager software version 3.1.1517.0823 (BioRad).

### Western blot analysis

Proteins were isolated following the RNA isolation according to the manufacturer’s recommendations (Peqlab). Proteins were dissolved in 1% SDS containing protease and phosphatase inhibitors, and protein content was measured with the bicinchoninic acid assay (Interchim, Montluçon Cedex, France). Western blot analysis was performed with specific antibodies as described previously (Fuchshofer et al. 2011,  2007). Specific antibodies were used as follows: goat anti-CTGF (1:500, Santa Cruz Biotechnology, Santa Cruz, USA), rabbit anti-α-tubulin (1:2500, Rockland Immunochemicals Inc., Gilbertsville, USA), donkey anti-goat (1:2000, Bethyl Laboratories Inc., Montgomery, USA), and goat anti-rabbit (1:5000, Cell Signaling Technology, Danvers, USA). Chemiluminescence was detected on a LAS 3000 imaging workstation (Fujifilm, Düsseldorf, Germany). For normalization of the signal intensity, α-tubulin was used as a loading control. The intensity of the bands detected by Western blotting was determined using appropriate software (AIDA Image analyzer software, Raytest, Straubenhardt, Germany).

### Microscopy and quantitative morphometry

For light and transmission electron microscopy, eyes were obtained from young adult animals at 9 weeks of age. Eyes were enucleated and fixed in Karnovsky’s solution (2.5% glutaraldehyde and 2.5% paraformaldehyde in 0.1 M cacodylate buffer) for 24 h (Karnovsky 1965). After rising in 0.1 M cacodylate buffer, postfixation was accomplished in a mixture of 1% OsO_4_ and 0.8% potassium ferrocyanide in 0.1 M cacodylate buffer for 2 h at 48 °C. After dehydration in a graded series of ethanol, the eyes were embedded in Epon (Serva, Heidelberg, Germany). Semithin sections (1 µm) were collected on uncoated glass slides and stained with methylene blue/azure II (Richardson et al. 1960). Ultrathin sections were mounted on uncoated copper grids, stained with uranyl acetate and lead citrate, and examined on a Zeiss Libra transmission electron microscope (Carl Zeiss AG). Quantitative morphometry of the retina was performed on semithin sections. The length of each retinal hemisphere was divided through 10, and the thickness at each point was measured. Means of the measured points were plotted in a spider diagram and statistical analysis was performed to compare single measure points of *CTGF*^*LacZ/*+^ and WT mice. Thickness of Descemet’s membrane was measured at 6 measure points in the peripheral cornea of semithin sections.

### Staining for β-galactosidase activity

Eyes of mice at different ages (postnatal days (P) 1, 5, 10, 15, 20, 49) were used. Embryos were obtained from timed matings with noon of the day of vaginal plug discovery designated as 0.5 days of gestation (E 0.5). *Ccn2/Ctgf* promotor activity was analyzed at E11.5 and E16.5. Eyes and embryos were fixed with 2% glutaraldehyde in 5 mM egtazic acid (EGTA; pH 7.3) and 2 mM MgCl_2_ dissolved in 0.1 M phosphate buffer for 30 min at RT. Followed by three washing steps with washing buffer (1 M MgCl_2_, 1% NaDC, 2% Tergitol in 0.1 M phosphate buffer), samples were immersed for 24 h at 37 °C in the dark with X-Gal staining solution (2 mM MgCl_2_, 0.01% sodium deoxycholate, 0.02% Nonidet-P40, 5 mM potassium ferrocyanide, 5 mM potassium ferricyanide, 1 mg/ml X-Gal dissolved in 0.1 M phosphate buffer) to visualize β-galactosidase activity. Afterwards, samples were washed three times for 10 min with washing buffer, followed by a second washing step for three times for 10 min with 0.1 M phosphate buffer. After incubation for 1 h with 50% isopropanol and 1 h with 70% isopropanol, samples were embedded in paraffin. Finally, 6 µm sections were cut on a Supercut microtom (Reichert-Jung, Kirchseeon, Germany).

### Immunohistochemistry

Eyes were enucleated and fixed in 4% (w/v) paraformaldehyde overnight or 2 h for retinal flat mount preparation. Eyes were equilibrated in 10%, 20%, and 30% sucrose, embedded in Tissue-Tek optimal cooling temperature compound (Sakura Finetek Europe B.V., Zoeterwounde, Netherlands) and stored at − 20 °C. Frozen 12 µm sections were cut on a cryostat. For retinal flat mounts, the whole retina was dissected and put on an object slide. Retinal flat mounts were pretreated with 50 mM NH_4_Cl (1 h, RT) and 0.5% Triton-X-100 (30 min, RT). Afterwards, sections and retinal flat mounts were incubated with 2% bovine serum albumin, 0.2% cold water fish gelatin, 0.1% Triton X-100 in 0.1 M phosphate buffer and stained with specific antibodies as follows: rabbit anti-β-galactosidase (1:100, ICL Lab, Portland, USA), chicken anti-GFAP (1:2000, LifeSpan Biosciences, Seattle, USA), goat anti-glutamine synthetase (1:100, Santa Cruz), goat anti-CD31 (1:100, R&D systems, Minneapolis, USA), goat anti-choline acetyltransferase (1:50, Chemicon International Inc., Temecula, USA), goat anti-Cy™3 IgG (1:2000, Jackson Research Laboratories, West Grove, USA), Alexa Fluor® 647 donkey anti-rabbit IgG (H+L) (1:200, Invitrogen), Alexa Fluor® 488 goat anti-chicken IgG (H+L) (1:1000, Invitrogen), Alexa Fluor® 488 donkey anti-goat IgG (H+L) (1:1000, Biotium Inc, Fremont, USA), biotinylated anti-rabbit IgG (H+L) (1:500, Vector Laboratories, Burlingame, USA), and Streptavidin Alexa Fluor® 488 (1:1000, Invitrogen). To control for unspecific binding of the secondary antibody, negative controls were performed in which primary antibodies had been omitted. Finally, 4,6-diamidino-2-phenylindoel (DAPI, Vector Laboratories) was added to counterstain nuclear DNA.

### Light and fluorescence microscopy

After immunohistochemical staining and staining for β-galactosidase activity, tissue sections and retinal flat mounts were analyzed using a Zeiss Axio Imager microscope (Carl Zeiss AG, Jena, Germany).

## Results

**Fig. 1:**
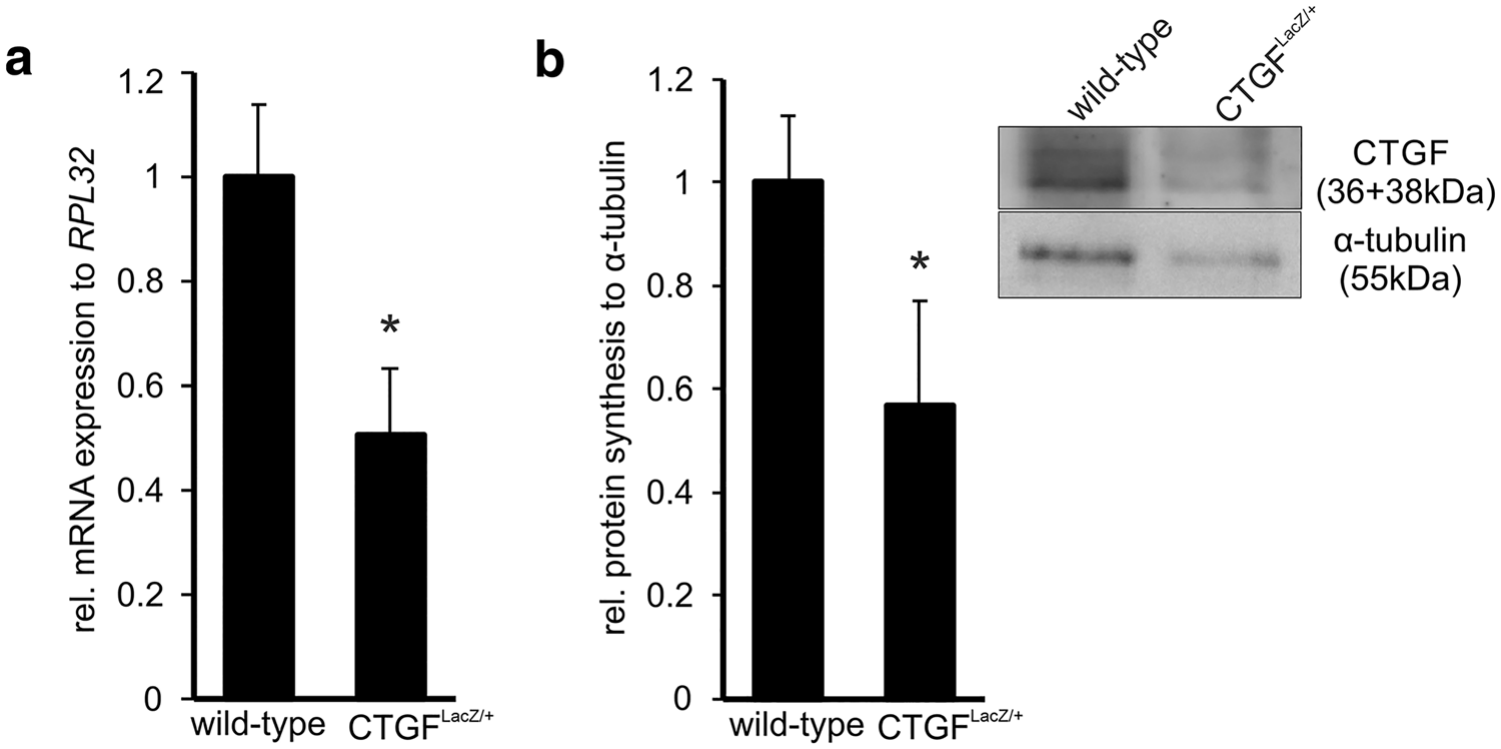
CCN2/CTGF and its mRNA in the sensory retina of *CTGF*^*LacZ/*+^ and wild-type mice at the age of 4 weeks. **a** Real-time RT-PCR for *Ccn2/Ctgf* mRNA in *CTGF*^*LacZ/*+^ mice compared to wild-type littermates (*p = 0.02). RPL32 was used as a housekeeping gene for analysis. **b** Western blot analysis for CCN2/CTGF in *CTGF*^*LacZ/*+^ mice when compared to wild-type controls (*p = 0.04). Two bands migrating at 36 kDa and 38 kDa are detected corresponding to the molecular weight of CCN2/CTGF and its modified form (Junglas et al. 2009). α-Tubulin was used for normalization of Western blot signal intensity. Mean values of wild-type controls were set at 1. Data represented as mean ± SEM

**Fig. 2:**
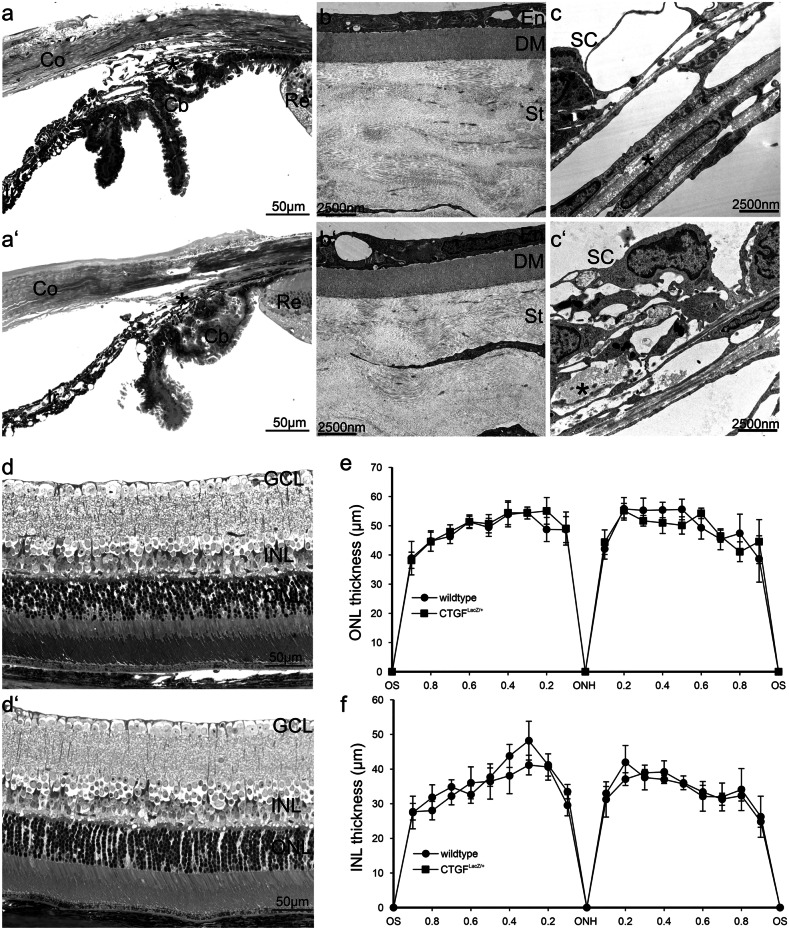
**a** Represented images of semithin sections of the iridocorneal angle and the cornea of WT mice **a** and *CTGF*^*LacZ/*+^ **a’** at the age of 9 weeks. There are no obvious morphological changes in *CTGF*^*LacZ/*+^ mice compared to control mice. **b** The ultrastructure of the cornea shows a regular orientation of the collagen bundles in the corneal stroma, the DM is composed entirely of electron dense material and with the characteristic banding pattern in the WT mice **b** and *CTGF*^*LacZ/*+^ **b’**. **c** The Schlemm’s canal endothelium and the juxtacanalicular trabecular meshwork showed the normal ultrastructural composition of the outflow tissues with a regular formed ECM (asterisk) in WT mice **c** and *CTGF*^*LacZ/*+^ **c’**. **d** Representative images of semithin sections of the central retina of WT mice **d** and *CTGF*^*LacZ/*+^ **d’** at the age of 9 weeks. There are no obvious morphological changes in *CTGF*^*LacZ/*+^ mice compared to control mice. The thickness of ONL **e** and INL  was quantified at 21 measure points on horizontal paraffin sections stretching from the nasal to temporal side through the ONH. The results are presented in spider diagrams. Data is represented as mean ± SEM, *n* = 3

To analyze the amounts of CCN2/CTGF in *CTGF*^*LacZ/*+^ mice with one null allele of *Ccn2/Ctgf*, we performed real-time RT-PCR and Western blot analyses using RNA or protein extracts isolated from the sensory retina of the mice at the age of 4 weeks. Analysis of retinal mRNA showed an approx. 50% reduction of *Ccn2/Ctgf* mRNA expression in *CTGF*^*LacZ/*+^ mice (0.50 ± 0.12, *p = 0.02, *n* = 4) when compared to their wild type (WT) littermates (1 ± 0.14, *n* = 3; Fig. 1a). The decrease in mRNA expression correlated with a decrease in the amounts of CCN2/CTGF which were significantly reduced (0.57 ± 0.35; *p = 0.04, *n* = 6) when compared to those of WT littermates (1 ± 0.23, *n *= 6; Fig. 1b). The lack of one functional *Ccn2/Ctgf* allele did neither cause obvious structural nor ultrastructural changes in young adult mice at 9 weeks of age (Fig. 2). More specifically, the cornea was completely transparent in all investigated animals. The corneal stroma of *Ccn2/Ctgf* mice did not differ from that of WT mice, neither in structure nor in amount of its collagen layers. (Fig. 2 a and a’; *CTGF*^*LacZ/*+^ *n* = 6; WT *n* = 5). Both in WT and mutant mice, Descemet’s membrane (DM) was entirely composed of electron dense material with its characteristic banding pattern (Fig. 2 b and b’). Thickness of Descemet’s membrane did not differ between *CTGF*^*LacZ/*+^ mice (2.26 ± 0.24 µm; *n* = 4) and their WT littermates (2.21 ± 0.09 µm, n = 2)*,* and was similar as observed by others in wild-type mice at the same age and with a similar genetic background (Jun et al. 2006). The inner surface of the cornea was entirely covered by endothelial cells and no signs of corneal vascularization were observed in any of the investigated animals (Fig. 2 a and a’; *CTGF*^*LacZ/*+^ *n* = 6; WT *n* = 5).

The anterior chamber angle was wide open, and trabecular meshwork and Schlemm’s canal were clearly visible in both groups (Fig. 2 a and a’). Moreover, the ultrastructural analysis of the conventional outflow pathways showed no structural differences between *CTGF*^*LacZ/*+^ mice and their WT littermates. Schlemm’s canal endothelium was normally formed showing giant vacuoles and the typical discontinuous basement membrane. In the juxtacanalicular region of the trabecular meshwork outflow pathways we observed in both groups regularly formed collagen fibrils and elastic fibers interrupted by optically empty spaces (Fig. 2 c and c’).

In both *CTGF*^*LacZ/*+^ mice and their WT littermates, the retina showed its normal layers (Fig. 2 d and d’). Moreover, the thicknesses of the outer nuclear layer and inner nuclear layer of *CTGF*^*LacZ/*+^ and WT littermates at the age of 2 months were not significantly different (*n* = 3) (Fig. 2 e and f). Taken together, reduction of CCN2/CTGF in heterozygous deficient *CTGF*^*LacZ/*+^ mice did not cause phenotypic changes, a finding that is in agreement with previous studies focusing on the retinal vasculature (Kuiper et al. 2007, 2008).

**Fig. 3:**
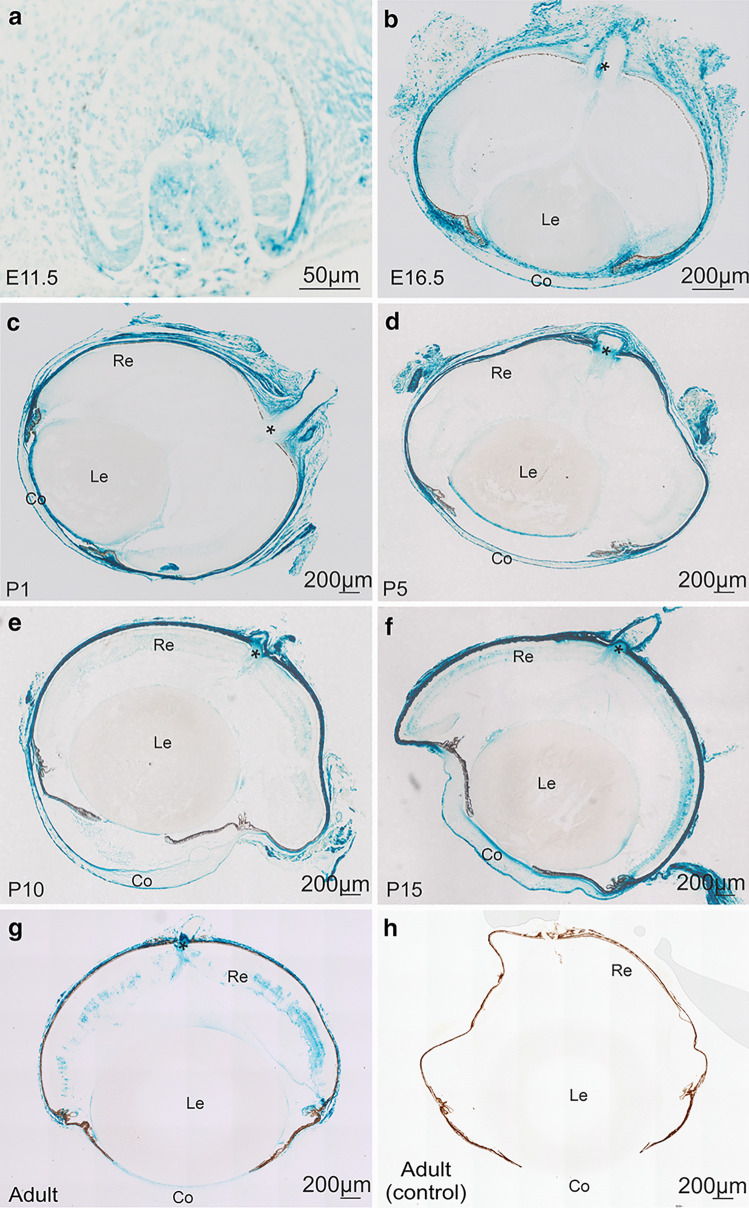
*Ccn2/Ctgf* promotor activity during embryonic and postnatal development of *CTGF*^*LacZ/*+^ mice. **a**, **b** Histological staining of β-galactosidase activity on embryonic day (E) 11.5 **a** and E16.5 **b** of the developing mouse eye of *CTGF*^*LacZ/*+^ mice. At E11.5, all cell types during formation of the lens vesicle and the optic cup, including those of the surrounding neural crest are positive for β-galactosidase. At E16.5, neural crest cells forming the sclera and the stroma of the developing choroid, ciliary body, and iris are labelled intensely. Additionally, the epithelium and endothelium of the cornea and the lens epithelium is positive for β-galactosidase. **c** At P1, the staining of β-galactosidase activity is similar compared to E16.5. **d** At P5, positive staining of β-galactosidase in the optic nerve is seen. **e** At P10, the first individual cells in the retina are positively stained and remain so until adulthood, while the staining in the sclera, cornea, and lens were barely detectable. **f**, **g** Throughout postnatal development and in adulthood, staining for β-galactosidase is intense in the stroma of the ciliary body and the adjacent tissue of the chamber angle including the trabecular meshwork. **h** Histological staining of β-galactosidase activity of wild-type littermates on the adult mouse eye shows no staining. Re retina, Le lens, Co cornea. *Optic nerve head. *n* ≥ 3

To analyze *Ccn2/Ctgf* promotor activity during embryonic development, we took advantage of the fact that in *CTGF*^*LacZ/*+^ mice, the β-galactosidase marker gene *LacZ* had been inserted into the open reading frame of *Ccn2/Ctgf*. At embryonic day (E) 11.5 during formation of the lens vesicle and the optic cup all cell types of the developing eye stained positive for β-galactosidase including those of the surrounding neural crest (Fig. 3a). At E16.5, the neural crest cells forming the sclera and the stroma of the developing choroid, ciliary body, and iris were labelled intensely for β-galactosidase (Fig. 3b). In addition, cells of both corneal epithelium and endothelium stained positive as did the cells of the lens epithelium. Positive staining was absent in lens fibers. Cells of the retinal pigment epithelium were essentially negative and the sensory retina showed only weak labeling in its anterior inner parts. Positive labeling continued to the inner epithelium of the optic cup anterior lip in region of the developing iris/ciliary body cells. At the developing optic nerve its surrounding meninges showed staining for β-galactosidase as did cells of the hyaloid artery passing through the optic nerve. Eyes of newborn mice (postnatal day (P1)) showed an essentially similar staining pattern (Fig. 3c). At P5, a distinct labeling was first observed at the optic nerve head that corresponded to the site of the developing glial lamina. Labeling of the glial lamina was also present at later stages and in adult eyes. A distinct staining of individual cells in the sensory retina became apparent at P10 and extended until adulthood (Fig. 3e–g). Simultaneously, the positive staining of sclera, cornea and lens became weaker and was barely detectable in adult eyes. Throughout postnatal life and in adulthood, staining for β-galactosidase was always intense in the stroma of the ciliary body and the adjacent tissues of the chamber angle including the trabecular meshwork (Fig. 3c–g). When control eyes of wildtype littermates where processed for β-galactosidase, no staining was detected (Fig. 3h).

### *Ccn2/Ctgf* expression in the anterior eye

**Fig. 4:**
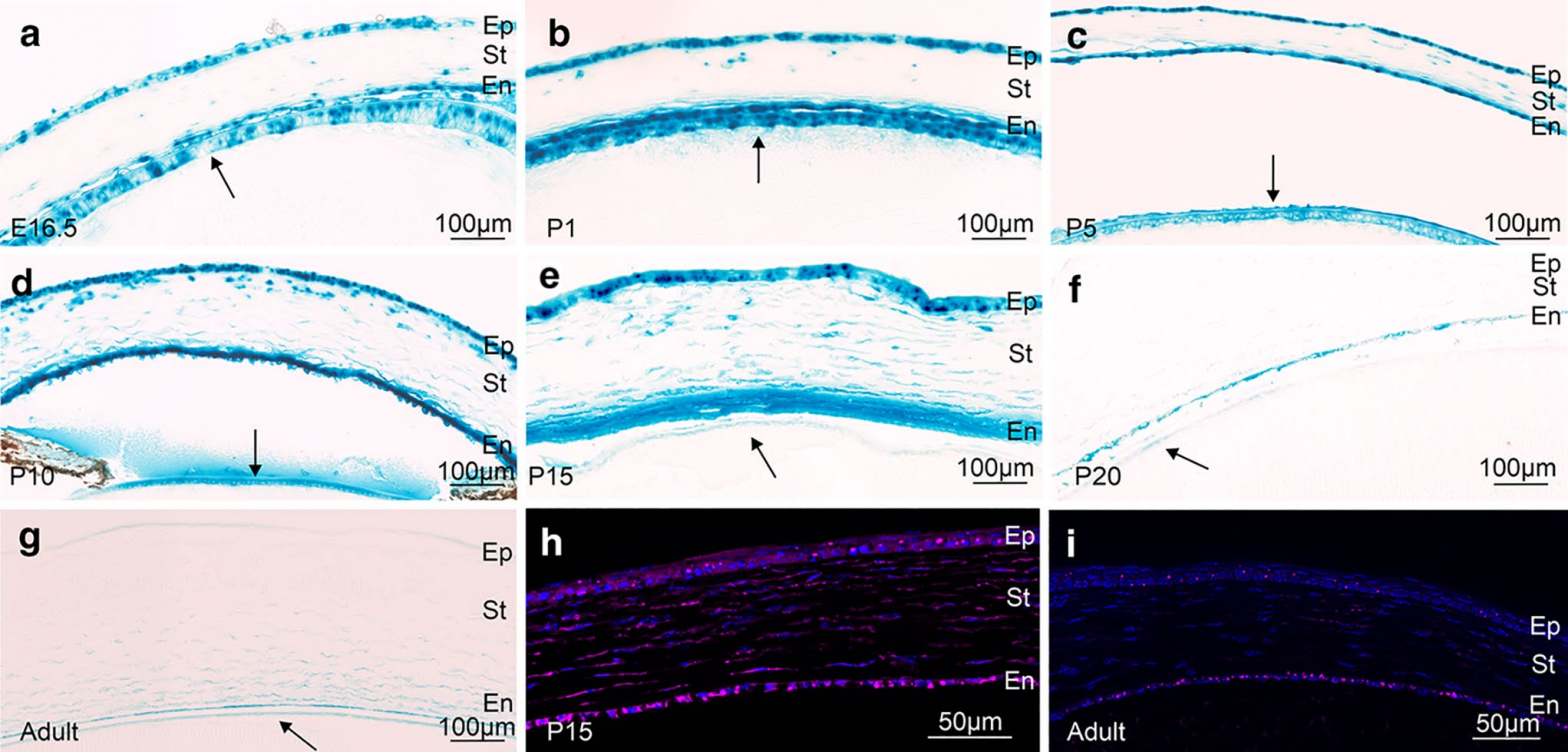
*Ccn2/Ctgf* promotor activity in the cornea and lens epithelium of *CTGF*^*LacZ/*+^ mice. Histological staining of β-galactosidase activity is intense from E16.5 to P10 in the epithelial layers of lens (arrow) and cornea, and in the corneal endothelium **a**–**d**. At P10 and P15, additional labeling is observed in keratocytes of the corneal stroma **d**, **e**. The staining of the lens epithelium is weaker from P15 to adulthood **e**–**g**. The same is the case for all corneal cell types at adulthood **g**. Immunohistochemical staining against β-galactosidase (purple) shows an intense immunoreactivity in the epithelium, endothelium, and stroma of the cornea at P15 **h**. β-Galactosidase immunoreactivity is much weaker in all corneal cell types in the adult eye **i**. Nuclei are stained with DAPI. Ep corneal epithelium, St corneal stroma, En corneal endothelium. Arrow indicates lens epithelium. *n* ≥ 3

Upon higher magnification of the developing cornea and lens, we observed that cells did not stain homogenously for β-galactosidase (Fig. 4a–g). In contrast, the staining was especially intense in distinct focal regions which we attributed to the fact that high amounts of the enzyme were present in the perinuclear endoplasmic reticulum causing local high enzymatic activity to process X-gal. Co-labeling with antibodies against β-galactosidase and DAPI to visualize nuclei showed perinuclear immunolabeling and supported our assumption (Fig. 4h, i). The staining pattern was intense from E16.5 to P10 in the epithelial layers of lens and cornea, and in the corneal endothelium (Fig. 4a–d). At P10 and P15, additional labeling was observed in keratocytes of the corneal stroma (Fig. 4d, e). Staining of the lens epithelium was much weaker when eyes from P15 to adulthood were investigated (Fig. 4e). The same was the case for all corneal cell types at P20 and later (Fig. 4f, g).

**Fig. 5:**
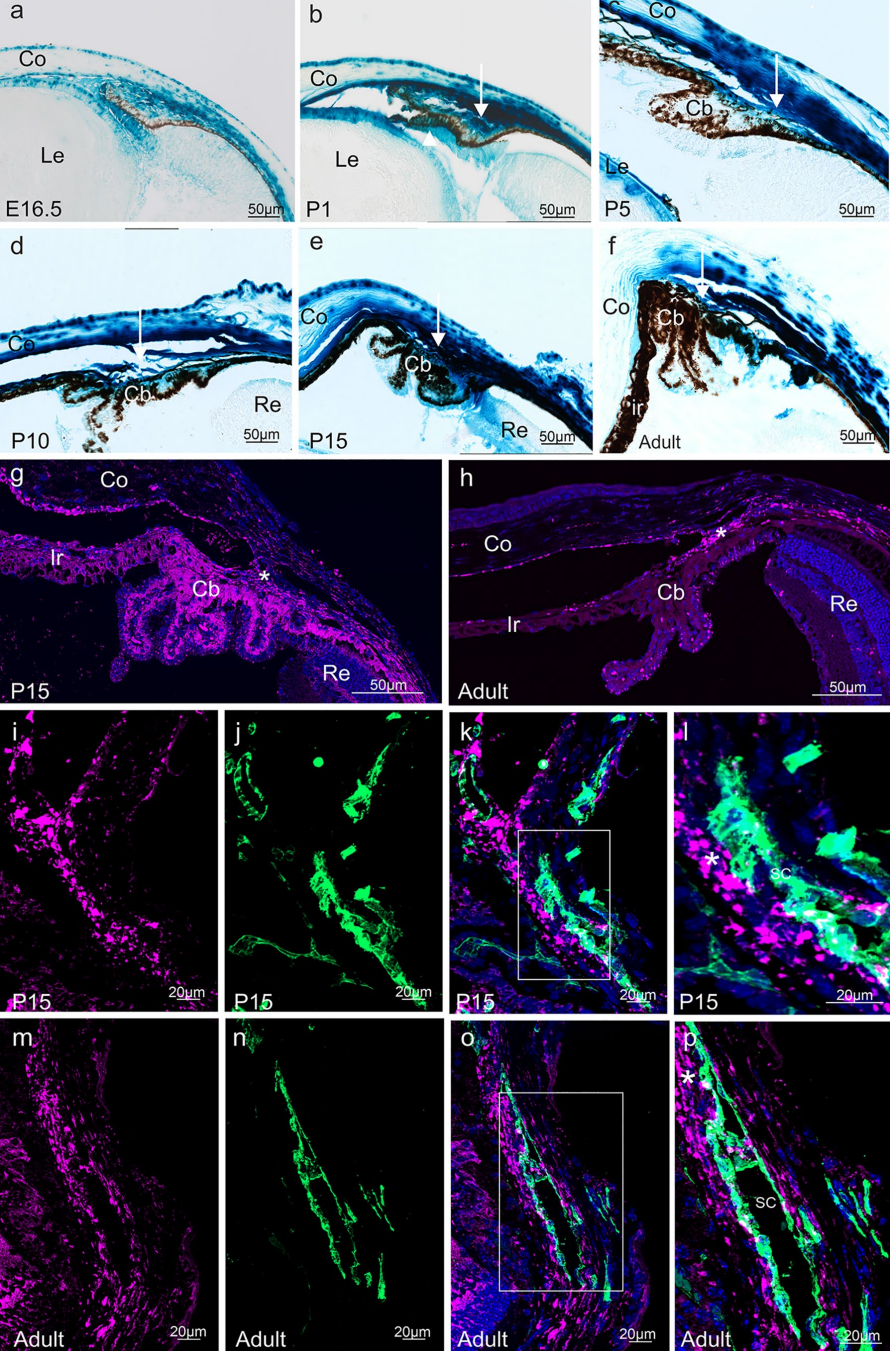
*Ccn2/Ctgf* promotor activity in the anterior eye segment of *CTGF*^*LacZ/*+^ mice. Histological **a**–**f** and immunohistochemical **g**, **h** β-galactosidase staining of the developing iridocorneal angle at E16.5, P1, P5, P10, P15, and the adult mouse eye. Histological staining for β-galactosidase activity is intense throughout postnatal development and in adulthood in the stroma of the ciliary body and the adjacent tissues of the chamber angle including the trabecular meshwork (arrow). Immunohistochemical staining against β-galactosidase (purple) shows the highest expression in the pigmented ciliary epithelium at P15. Furthermore, stroma cells of iris/ciliary body and adjacent choroid, chamber angle, and corneal endothelium express β-galactosidase. Immunoreactivity is also detected in cells of the limbus region. In the adult eye, immunoreactivity of β-galactosidase is markedly weaker in cells of the anterior uvea and ciliary epithelium but remains intense in the region of the trabecular meshwork outflow pathways (asterisk). Nuclei are stained with DAPI. **i**–**p** Immunohistochemical staining against β-galactosidase (purple) and CD31 (green) identifies cells in the inner wall endothelium of Schlemm’s canal as a source of the *Ccn2/Ctgf* promotor activity at P15 **i**–**l** and in the adult eye **m**–**p**. Nuclei are stained with DAPI. Co cornea, Ir Iris, Cb ciliary body, Re retina, SC Schlemm’s canal. *n* ≥ 3

In the chamber angle region, positive labelling of the stroma in the developing iris/ciliary body was intense at E16.5 and remained so in postnatal life until adulthood (Fig. 5a–f). Between P5 and 15, there was an additional very intense labeling in the limbus area marking the transition between cornea and sclera (Fig. 5c–e). Since during that time period the trabecular meshwork outflow pathways including Schlemm’s canal and its collector channels develop in the mouse eye (Cvekl and Tamm 2004), we aimed at identifying more about the nature of the β-galactosidase-positive cells in this region. To this end, we again performed immunohistochemistry as the blue reaction product of the β-galactosidase stain tends to diffuse in regions of high *LacZ* expression. By using immunolabeling, we identified as site of the highest expression in the P15 anterior eye the pigmented ciliary epithelium (Fig. 5g). In addition, we confirmed that the neural crest-derived cells of the anterior eye such as stroma cells of iris/ciliary body and adjacent choroid, chamber angle and corneal endothelium express β-galactosidase (Fig. 5g). Corroborating our findings seen after X-gal histochemistry, immunoreactivity for β-galactosidase was detected in the cells of the limbus region. In the adult eye, β-galactosidase immunoreactivity was markedly weaker in cells of anterior uvea and ciliary epithelium but remained intense in region of the trabecular meshwork outflow pathways (Fig. 5h). To identify which cell type of the outflow pathways expresses β-galactosidase, we performed double-immunohistochemistry with antibodies against β-galactosidase and CD31 (Fig. 5i–p) that bind to vascular endothelial cells including those of Schlemm’s canal (Herrnberger et al. 2012). Both at P15 (Fig. 5i–l) and in the adult eye (Fig. 5 m–p), cells of the trabecular meshwork adjacent to the inner site of Schlemm’s canal showed intense immunoreactivity. In addition, some cells in the inner wall endothelium of Schlemm’s canal were positively labeled (Fig. 5i–p).

### *Ccn2/Ctgf* expression in the posterior eye

**Fig. 6:**
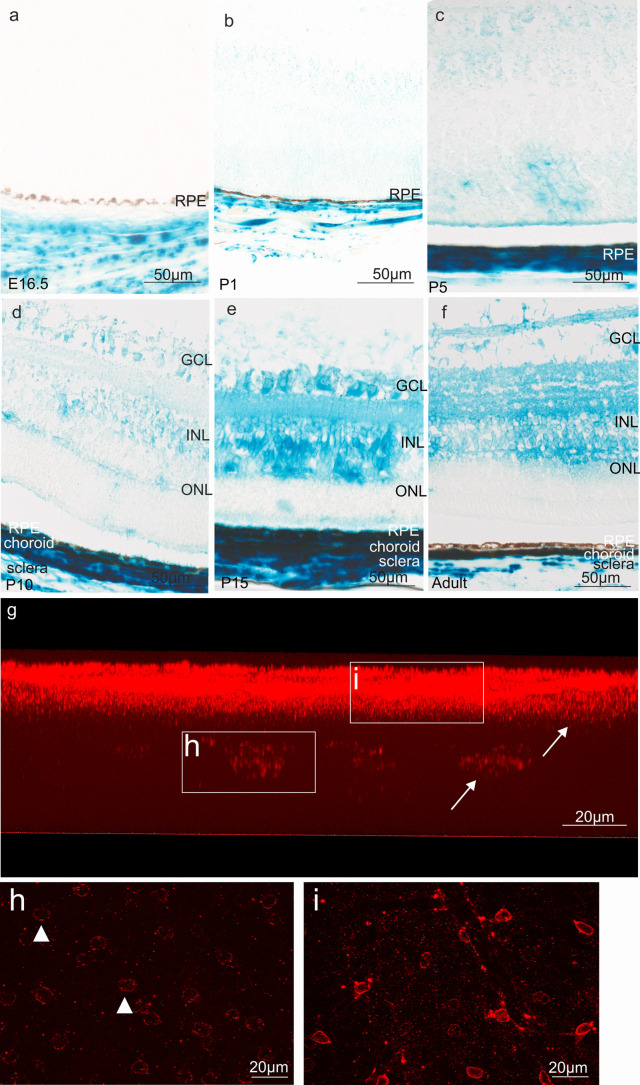
*Ccn2/Ctgf* promotor activity in the retina of *CTGF*^*LacZ/*+^ mice. **a–f** Histological staining of β-galactosidase activity of the retina from E16.5 to adulthood. A distinct staining of individual cells in the sensory retina is apparent at P10 and increases in intensity and extent until adulthood. GCL ganglion cell layer, INL inner nuclear layer, ONL outer nuclear layer, RPE retinal pigmented epithelium. **g**–**i** Immunohistochemical staining against β-galactosidase (red) of a retinal flat mount of *CTGF*^*LacZ/*+^ mice, represented in a vertical view of the 3D reconstruction of z-stacks through the entire retina. **i** Single recording of the ganglion cell layer shows the staining against β-galactosidase (red) in the vessels of the superficial plexus and adjacent cells (arrow). **h** Single recording of the inner nuclear layer identifies another cell type (arrowhead) with immunoreactivity against β-galactosidase (red). *n* ≥ 3

**Fig. 7:**
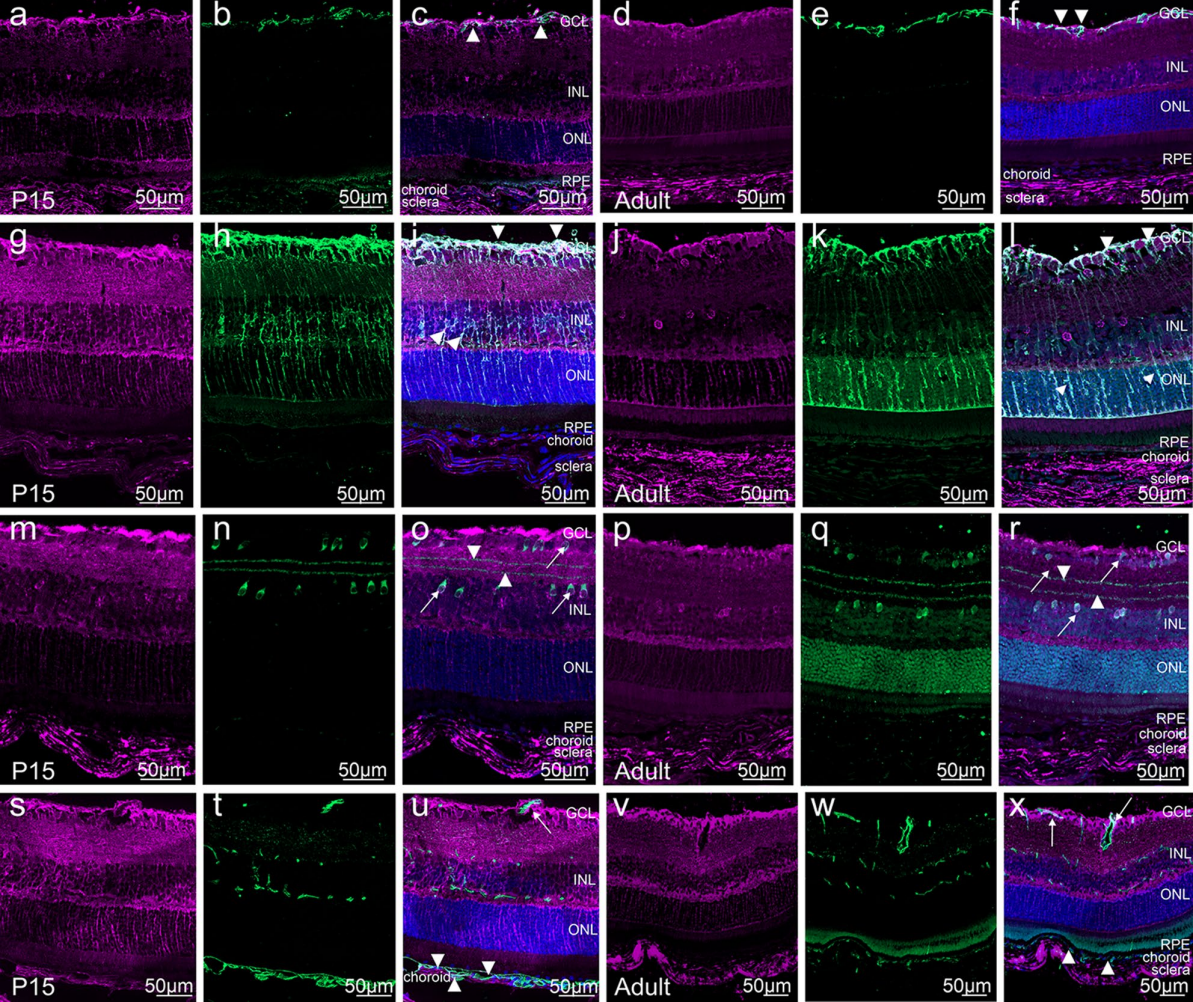
Cell type specific *Ccn2/Ctgf* promotor activity in the retina of *CTGF*^*LacZ/*+^ mice. Immunohistochemical staining against β-galactosidase (purple) and different retinal cell type markers (green). **a**–**f** Double-immunohistochemistry against β-galactosidase and GFAP shows weak immunoreactivity for β-galactosidase in retinal astrocytes (arrowhead) on P15 or adulthood. **g**–**l** Double-immunohistochemistry against β-galactosidase and glutamine synthetase (GS) shows an intense colocalization in the Müller cells processes (lower arrowheads) and endfeet (upper arrowheads) at P15 and in the adult retina. **m**–**r** Double-immunohistochemistry against β-galactosidase and choline acetyltransferase (ChAT) shows colocalization of β-galactosidase in starburst amacrine cells in the INL (arrow) and displaced starburst amacrine cells in the GCL (arrow) at P15 and in the adult retina. **s**–**x** Double-immunohistochemistry against β-galactosidase and CD31. CD31 immunoreactivity identifies the endothelial cells of the three retinal vascular plexus and the choriocapillaris. β-Galactosidase immunoreactivity is seen in the endothelial cells of the retinal plexus (arrow) and choroidal vasculature (arrowhead) in the P15 and adult retina. GCL ganglion cell layer, INL inner nuclear layer, ONL outer nuclear layer, RPE retinal pigmented epithelium, β-Gal β-galactosidase. Nuclei were stained with DAPI. *n* ≥ 3

At E16.5 and P1 β-galactosidase labeling was observed in the developing choroid and sclera, while the retinal pigment epithelium and the sensory retina were negative (Fig. 6a, b). At P5-positive staining became detectable in the neuroblastic layer of the developing sensory retina and, albeit with weaker intensity, in the ganglion cell layer (Fig. 6c). Staining of the ganglion cell and of the inner nuclear layer became more distinct at P10 (Fig. 6d). An intense signal was observed in both layers at P15 and in adulthood (Fig. 6e, f). From that time on until adulthood, staining was seen in the inner and outer plexiform layers (Fig. 6e, f). Positive staining in the choroid was seen through all postnatal stages until adulthood (Fig. 6b–f). To obtain more information on the specific cell type that expresses β-galactosidase in the ganglion cell and inner nuclear layer, we immunostained retinal flat mounts of *CTGF*^*LacZ/*+^ mice with antibodies against β-galactosidase and investigated a vertical view of 3D reconstructions of z-stacks through the entire retina (Fig. 6g–i). Again, we observed intense immunolabeling in ganglion cell layer and inner nuclear layer corroborating our findings obtained through X-gal staining. When analyzing individual stacks in the ganglion cell layer in a horizontal view, we identified positively labelled cells with cellular processes that were in contact with vessels on the retinal surface and very likely represented astrocytes (Fig. 6i). In contrast, cells in the inner nuclear layer had no obvious association with retinal capillaries (Fig. 6h). Next, we analyzed meridional sections through the retina that were again immunostained for β-galactosidase. Comparable to findings obtained with X-gal staining, immunoreactivity was seen in inner and outer plexiform layer, inner nuclear layer and ganglion cell layer. Double-immunostaining with antibodies against GFAP to visualize astrocytes on the retinal surface distinctly overlapped with β-galactosidase immunoreactivity and supported our assumption that retinal astrocytes express *Ccn2/Ctgf* (Fig. 7a–f). Next, double-immunostaining with antibodies against glutamine synthetase (GS) to stain Müller cells and β-galactosidase were performed, which showed a clear *Ccn2/Ctgf* expression in Müller cell processes and endfeet (Fig. 7g–l). Furthermore, we observed some scattered β-galactosidase-positive cells scattered in the ganglion cell layer and in the inner nuclear layer, which were negative for GS and GFAP. The localization and appearance of the cells matched with the description of displaced amacrine cells. The majority of displaced amacrine cells are starburst amacrine cells (Perez De Sevilla Muller et al. 2007), which is why we performed double-immunohistochemistry with antibodies against choline acetyltransferase (ChAT), a specific marker for starburst amacrine cells and against β-galactosidase. As expected, ChAT-immunoreactive somata were localized to the inner portion of the inner nuclear layer and the outer border of the inner plexiform layer, both at P15 and in adulthood (Fig. 7m–r). Positive staining for β-galactosidase was seen in their perinuclear cytoplasm (Fig. 7m–r). Finally, we performed double-immunohistochemistry for β-galactosidase and CD31 to identify that endothelial cells of choroidal and retinal vessels homogenously express *Ccn2/Ctgf* (Fig. 7s–x).

### Ccn2/Ctgf expression in the optic nerve

**Fig. 8:**
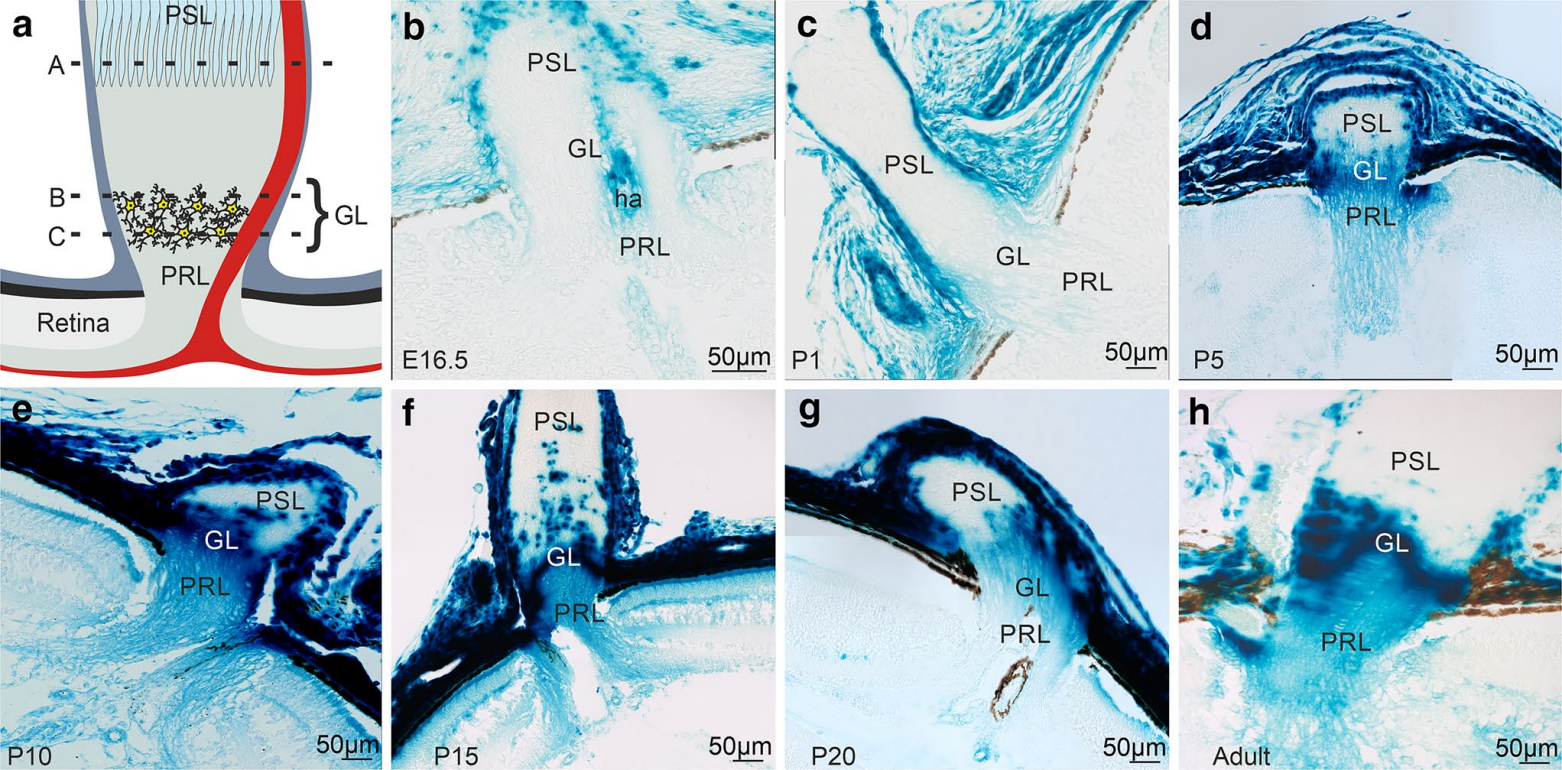
*Ccn2/Ctgf* promotor activity in the optic nerve of *CTGF*^*LacZ/*+^ mice. **a** Schematic of the optic nerve head showing the major elements in this region. Different positions indicated with A–C depict represent the positions where cross sections were performed shown in Fig. 9. **b** At E16.5, the meninges of the developing optic nerve and the cells of the hyaloid artery show positive staining of β-galactosidase activity. **c** At P1, a similar staining pattern is seen. **d** At P5, a distinct labeling at the optic nerve head that corresponds to the site of the developing glial lamina is observed. **e–h** From P10 to adulthood, an intense staining of the glial lamina and a weaker signal in the prelaminar region is present. At P15, several distinct spots are intensively labeled in the postlaminar region of the optic nerve. PSL postlaminar region, GL glial lamina, PRL prelaminar region, ha hyaloid artery. *n* ≥ 3

**Fig. 9:**
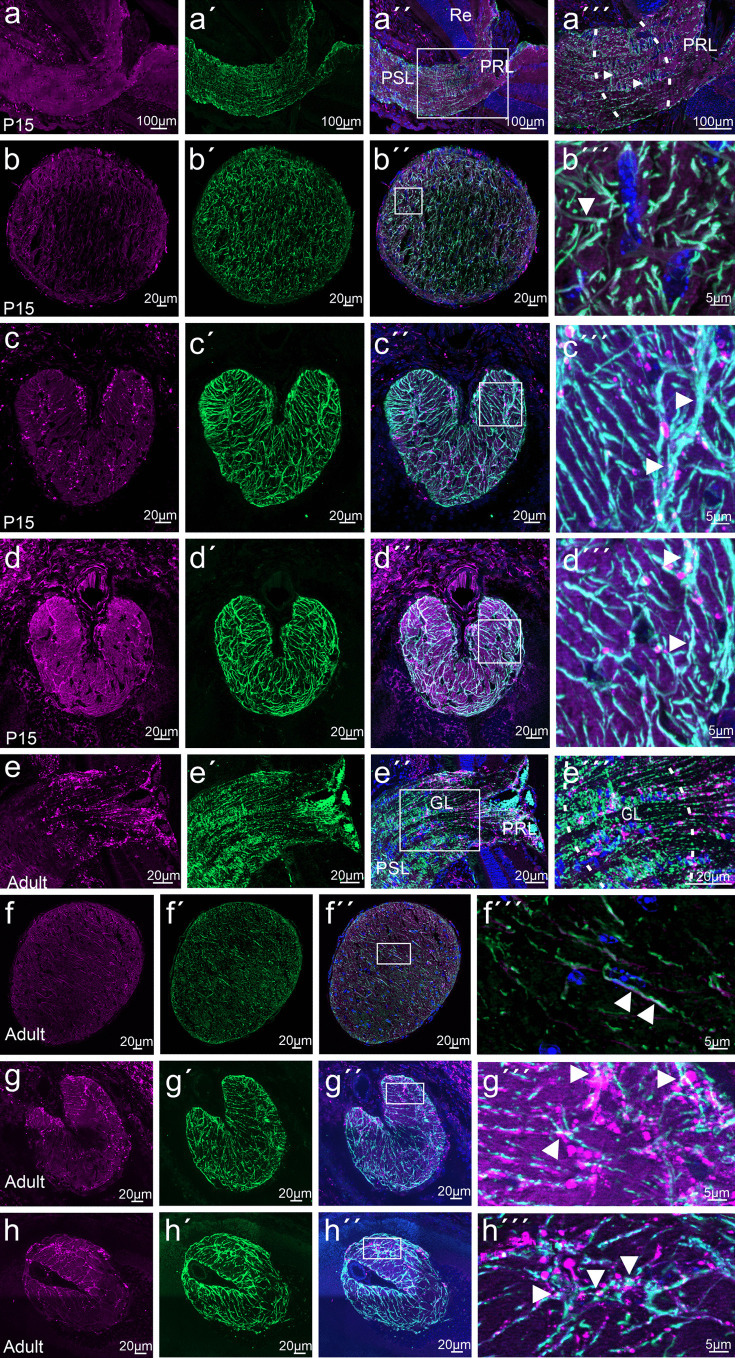
Cell type specific *Ccn2/Ctgf* promotor activity in the optic nerve of *CTGF*^*LacZ/*+^ mice. **a**,** e** Double-immunohistochemical staining against β-galactosidase (purple) and GFAP (green) on sagittal sections of the optic nerve at P15 **a** and adult mouse eye **e**. β-Galactosidase immunoreactivity is seen in region of the glial lamina and the prelaminar region that colocalizes with GFAP positive astrocytes (higher magnification arrowhead). Dotted line in enlarged merged image represents the region of the glial lamina. **b–d**,** f–h** Double-immunohistochemical staining against β-galactosidase and GFAP on cross sections of the optic nerve at P15 and the adult mouse eye. Schematic in Fig. 8a depicts the different positions (A–C), where sections were performed within the optic nerve. **b**,** f** In position A, the postlaminar, myelinated region of the optic nerve is seen in cross section. Immunoreactivity for β-galactosidase and GFAP is weak in the postlaminar region in the optic nerve. Position B **c**, **g** and C **d**, **h** localization of cross sections through the glial lamina. Double-immunostaining shows a close association and partial overlapping of β-galactosidase with GFAP-labelled astrocytes (higher magnification arrowhead). Nuclei were stained with DAPI. *n* ≥ 3

Until P1, the optic nerve and the optic nerve head remained unstained for β-galactosidase, with the exception of the hyaloid artery and its surrounding meninges that were positively labeled (Fig. 8b, c). At P5, positive labeling for β-galactosidase could be observed in the glial lamina of the optic nerve head. The staining was very intense at P10 and remained so until adulthood (Fig. 8e–h). In addition, from P5 until adulthood there was positive labeling of the optic nerve head prelaminar region albeit with less intensity than in the glial lamina (Fig. 8d–h). At P15, several distinct spots were intensively labeled in the postlaminar region of the optic nerve (Fig. 8f), a staining pattern that was less prominent at P20 and in the adult optic nerve. To identify the cellular origin of *Ccn2/Ctgf* promoter activity at the optic nerve head, we performed double-immunohistochemistry with antibodies against both β-galactosidase and GFAP which is highly expressed in astrocytes of the mouse glial lamina (Howell et al. 2007; Sun et al. 2009). Immunoreactivity for β-galactosidase and GFAP was weak in the postlaminar region of the P15 optic nerve but more intense in the posterior part of the glial lamina (Fig. 9a–d). In the anterior glial lamina, there was intense immunoreactivity for β-galactosidase corroborating the findings seen with X-gal staining (Fig. 9c, d). Higher magnification showed a close association and partial overlapping with GFAP labelled astrocytes. We attributed lack of complete overlap to the different intracellular location of GFAP-positive intermediate filaments and perinuclear β-galactosidase. The staining pattern in the adult optic nerve head was essentially similar to that seen in P15 (Fig. 9e–h).

## Discussion

We conclude that CCN2/CTGF signaling is involved in the processes that govern neural crest (NC) morphogenesis during late embryonic and postnatal eye development. In the adult eye, it primarily contributes to functional processes in the trabecular meshwork outflow pathways, the choroidal and retinal vasculature, and the optic nerve head. This conclusion rests on our observation of (1) *Ccn2/Ctgf* promoter activity in NC-derived cells differentiating to uveal and corneal tissues, and (2) persistent strong promoter activity in trabecular meshwork, endothelial cells of Schlemm’s canal, retinal and choroidal vessels, and astrocytes of the optic nerve head glial lamina.

NC cells migrate to the developing eye to form the corneal stroma and endothelium, and later during a second migration wave the stroma of choroid, iris and ciliary body, and the trabecular meshwork (Cvekl and Tamm 2004). NC migration in cranial development is under control of a variety of transcription factors that initiate distinct signaling processes to guide NC migration and differentiation. There is substantial evidence obtained from studies of a variety of induced mutant mouse strains that members of the TGF-β superfamily play a critical role in those signaling processes (Conway and Kaartinen 2011; Oka et al. 2007). The same appears to be the case for NC cells that are involved in eye development as both deficiency in TGF-β (Iwao et al. 2009; Saika et al. 2001; Sanford et al. 1997) or BMP4 signaling (Chang et al. 2001), or overexpression of TGF-β signaling (Flügel-Koch et al. 2002) interfere with NC-derived ocular morphogenesis. The well-documented amplifying activity of CCN2/CTGF on TGF-β signaling (Abreu et al. 2002; Gressner et al. 2009; Khankan et al. 2011; Mori et al. 1999) may well be involved in the action of TGF-β signaling on cranial NC development. In support of this assumption are observations that CCN2/CTGF can rescue the phenotype caused by deficient TGF-β signaling in the developing cranial NC such as the formation of mandibular dysgenesis or cleft palate (Oka et al. 2007), or causes similar defects when deleted (Ivkovic et al. 2003; Lambi et al. 2012; Tarr et al. 2017). Since our findings show high *Ccn2/Ctgf* expression in ocular NC cells, it is tempting to speculate that it plays a similarly important role to modulate NC morphogenesis during eye development. The availability of CCN2/CTGF during those critical processes in eye development may not exclusively depend on autocrine secretion by NC cells. In contrast, it may well be supported by adjacent paracrine sources as we also detected *Ccn2/Ctgf* promoter activity in immediately adjacent cells of non-NC origin such as lens epithelium or pigmented ciliary epithelium.

We observed that the *Ccn2/Ctgf* promoter activity in NC-derived ocular tissues might be depended on developmental stages. In the adult eyes, the *Ccn2/Ctgf* promoter activity remained mainly detectable in the trabecular meshwork and the adjacent limbus. This observation agrees with data obtained through the NEIBank sequence tag analysis that identified *Ccn2/Ctgf* as one of the highest expressed genes in the human trabecular meshwork (Tomarev et al. 2003). We showed in previous work that CCN2/CTGF is critically required for actin stress fiber formation and contractility of trabecular meshwork cells and is simultaneously involved in the modulation of their ECM deposition (Junglas et al. 2012,  2009; Kuespert et al. 2015). Since both processes are critically involved in generating and modulating aqueous humor outflow resistance (Stamer et al. 2015; Tamm et al. 2015), the continuous high promoter activity of *Ccn2/Ctgf* in the mouse eye is not necessarily surprising. An interesting new aspect of our study is that we were able to show promoter activity in Schlemm’s canal cells in situ corroborating data from previous in vitro studies (Overby et al. 2014).

Schlemm’s canal cells are not the only type of vascular endothelial cells in the eye that display *Ccn2/Ctgf* promoter activity as our data show that the same is the case for retinal and choroidal endothelial cells. *Ccn2/Ctgf* promoter activity or mRNA expression has also been shown for developing and adult vascular endothelial cells of other organs (Friedrichsen et al. 2005, 2003; Hall-Glenn et al. 2012). Our findings in the eye agree with those of Pi and colleagues (Pi et al. 2011) who observed CCN2/CTGF immunostaining in retinal vascular endothelial cells and pericytes during retinal angiogenesis. In addition, the authors showed that CCN2/CTGF is critically required for retinal angiogenesis, since the injection of antibodies against CCN2/CTGF into the vitreous of P2 mice resulted in a decrease of the superficial retinal vascular plexus. The continuous promoter activity of *Ccn2/Ctgf* in the adult mouse eye agrees with data obtained by immunohistochemistry of human eyes (van Setten et al. 2016) and strongly indicates a function for the adult eyes that is beyond angiogenesis in retinal development. A potential function could be related to the observation that *Ccn2/Ctgf* expression is upregulated by vascular endothelial growth factor (VEGF) (He et al. 2003; Suzuma et al. 2000) and its known inhibiting role of VEGF-induced angiogenesis by formation of VEGF-CTGF complexes (Inoki et al. 2002; Jang et al. 2004). The continuous expression of CCN2/CTGF in adult retinal vessel might be useful to antagonize signaling processes that have the potential to cause retinal neovascularization and its associated adverse effects on retinal structure and function. An additional paracrine expression of *Ccn2/Ctgf *in perivascular astrocytes on the retinal surface may well contribute to this function. Quite similarly Müller cell-derived CCN2/CTGF likely antagonizes a VEGF-induced growth signal on intraretinal capillaries while the continuous *Ccn2/Ctgf *expression in adult choroidal vessels likely contributes to prevent choroidal neovascularization. Overall, the TGF-β amplifying function of CCN2/CTGF is likely to contribute to its effects on retinal and choroidal vessels as lack of TGF-β signaling in the eye causes retinal and choroidal neovascularization that finally destroys retinal structure and function (Braunger et al. 2015b; Schlecht et al. 2017).

An unexpected finding was the very high expression of *Ccn2/Ctgf* in the glial lamina of the mouse optic nerve head. This area is equivalent to the *Lamina cribrosa* in the primate eye (Stowell et al. 2017; Tamm et al. 2017) and contains a dense network of intertwined astrocytes that constitutively express GFAP and show other similarities to white matter fibrous astrocytes (Sun et al. 2009). Unlike their counterpart in the brain, astrocytes in the mouse glial lamina are under a substantial and continuous mechanical strain induced by intraocular pressure (IOP) as are the astrocytes in the *Lamina cribrosa* of the primate eye. IOP is the most critical risk factor for damage of axons at the optic nerve head in glaucoma, both in the primate and the mouse eye (Quigley 2011; Steinhart et al. 2014; Weinreb et al. 2014). Experimental data indicate a rapid reorganization of glial lamina astrocytes and their processes in response to brief and mild elevations of IOP (Sun et al. 2013). A signaling factor like CCN2/CTGF that is known to modulate the actin cytoskeleton and the synthesis of fibrillar ECM components that are connected via integrins may be an essential requirement for quick astrocyte reorganization in response to mechanical stimuli. In support of this assumption, we observed the promoter activity in the cornea and sclera only during development, which might be necessary to build up the ECM of these load-bearing structures. The slow ECM turnover in the cornea and sclera in the adult tissues might not require CCN2/CTGF activity under normal conditions. In contrast, in tissues that are subject to continuous micro-strain, CCN2/CTGF might be induced through mechanical stimuli. This scenario likely involves activation of mechanotransducing proteins such as YAP and TAZ, which were both identified in human trabecular meshwork cells and in fibroblasts (Chudgar et al. 2006; Guo et al. 2011; Schild and Trueb 2002; Thomasy et al. 2013). There is substantial evidence that CCN2/CTGF inhibits oligodendrocyte differentiation in vitro and in vivo (Lamond and Barnett 2013; Stritt et al. 2009). In the brain, it is neuronal-derived CCN2/CTGF that acts negatively on myelination (Ercan, et al. 2017). It is tempting to speculate that in the optic nerve head, the local high amounts of astrocyte-derived CCN2/CTGF inhibit myelination of the optic nerve axons that pass through the glial lamina and that the lack of myelination increases their vulnerability to mechanical strain induced by intraocular pressure. Further studies using mice with an induced conditional deletion of *Ccn2/Ctgf* appear to be the appropriate tool to shed light on the distinct functional role(s) of *Ccn2/Ctgf* in the glial lamina of the optic nerve head.
